# Treatment of Nanocellulose by Submerged Liquid Plasma for Surface Functionalization

**DOI:** 10.3390/nano8070467

**Published:** 2018-06-26

**Authors:** Denis Mihaela Panaitescu, Sorin Vizireanu, Cristian Andi Nicolae, Adriana Nicoleta Frone, Angela Casarica, Lavinia Gabriela Carpen, Gheorghe Dinescu

**Affiliations:** 1 Department of Polymer, National Institute for Research and Development in Chemistry and Petrochemistry, 202 Spl. Independentei, 060021 Bucharest, Romania; cristian.nicolae@icechim-pd.ro (C.A.N.); ciucu_adriana@yahoo.com (A.N.F.); 2 National Institute for Laser, Plasma and Radiation Physics, Atomistilor 409, Magurele-Bucharest, 077125 Ilfov, Romania; lavinia.carpen@infim.ro (L.G.C.); dinescug@infim.ro (G.D.); 3 National Institute for Chemical—Pharmaceutical Research and Development, 112 Calea Vitan, 031299 Bucharest, Romania; angelacasarica@yahoo.com

**Keywords:** nanocellulose, plasma treatment, dielectric barrier discharge, submerged liquid plasma, polymer nanocomposite

## Abstract

Tailoring the surface properties of nanocellulose to improve the compatibility of components in polymer nanocomposites is of great interest. In this work, dispersions of nanocellulose in water and acetonitrile were functionalized by submerged plasmas, with the aim of increasing the quality of this reinforcing agent in biopolymer composite materials. Both the morphology and surface chemistry of nanocellulose were influenced by the application of a plasma torch and filamentary jet plasma in a liquid suspension of nanocellulose. Depending on the type of plasma source and gas mixture the surface chemistry was modified by the incorporation of oxygen and nitrogen containing functional groups. The treatment conditions which lead to nanocellulose based polymer nanocomposites with superior mechanical properties were identified. This work provides a new eco-friendly method for the surface functionalization of nanocellulose directly in water suspension, thus overcoming the disadvantages of chemical treatments.

## 1. Introduction

Nanocellulose (NC) is a very attractive material due to its unique properties, i.e., lightweight, strength, flexibility, biodegradability and biocompatibility, wide availability and cost efficiency of the sources [1,2,3]. The increasingly severe environmental policies and the exceptional properties of NC promote it as a valuable material for a wide range of applications. NC can be extracted from a multitude of primary sources, such as wood, plants, algae, and sea animals (tunicates) as well as from secondary sources or wastes resulting from the industrial processing of wood, bast fiber crops, vegetables or fruits, etc. [3,4,5,6,7,8]. In order to obtain NC, mechanical, chemical, enzymatic processes or a combination of the above are, generally, used. A promising approach to prepare NC is from bacterial cellulose (BC), which is biosynthesized by some bacteria as an extracellular material [8,9,10,11,12,13]. By applying mechanical disintegration [8,10,11] or chemical treatments (acid hydrolysis, 2,2,6,6-tetramethylpiperidine-*N*-oxyl (TEMPO) catalyzed oxidation) [9,12,13], BC membranes may release nanofibers. 

For most applications, the surface properties of cellulose must be improved by physical or chemical treatments. Although BC has OH groups on the surface, which may react with other groups, it is not easy to be functionalized. This is caused by its high purity compared to plant cellulose which undergoes multiple treatments to remove other components (lignin, hemicellulose, pectin) and its high crystallinity (generally over 80% [10]). For example, the morphology and arrangement of BC nanofibers in the 3D structure of BC membranes were not changed by TEMPO oxidation [14] and a lower rate was noticed in the TEMPO-mediated oxidation of BC compared to cellulose from other sources [13]. 

Several attempts to surface functionalize BC by chemical routes have been reported [15,16,17,18]. Wet BC membranes were grafted with (3-aminopropyl)triethoxysilane (APTES) in ethanol and showed increased hydrophobicity and antibacterial properties [15]. However, a slight decrease of cell viability was reported for BC-g-APTES membranes with the increase of grafting yield [15]. Dried BC membranes were grafted with two organosilanes, octadecyltrichlorosilane and APTES in hexane showing increased hydrophobicity in the first case and improved cell attachment and spreading in the case of APTES treatment [16]. Carboxymethylated cellulose was obtained starting from water suspension of BC, previously homogenized with a blender, which was solvent-exchanged to isopropanol and carboxymethylated [18]. The degree of substitution was tailored by controlling the reaction conditions. 

It is worth noting that the use of chemical solvents and reagents to adjust the properties of BC may remove some of its above-mentioned benefits and may also raise environmental issues. Therefore, plasma treatments are more suited when food industry and biomedical applications are foreseen. Cold plasma is now seen as an environmentally safe and more efficient technique compared to “chemical” processing for many fields, especially for textile, food packaging, and biomedicine. 

Plasma treatment of cellulose has been intensively studied for the textile and paper industry and other fields, for different purposes like cleaning, sterilization, activation, increased hydrophilicity or hydrophobicity [19,20,21,22,23,24,25]. Plasma pretreatment is very efficient for the activation of textile surfaces [19,20]. For example, the surface of cellulose-based textiles was modified using nitrogen plasma which ensured new active and binding sites for the attachment of silver nanoparticles [19]. Plasma and silver treated textiles showed good antibacterial activity even after 15 laundering cycles [19]. Plasma treatment with a helium/oxygen mixture was applied to grey cotton knitted fabric to remove surface impurities [20]. The plasma treatment was efficient in cleaning the textile and enhancing the wettability but the yellowness was not reduced probably because of the thermal oxidation. Recent study has shown that the microwave Ar plasma conditions influenced the properties of cotton fabric in different directions, i.e., applying maximum gas pressure and minimum power led to increased hydrophobicity and crystallinity and a smooth surface [21]. High hydrophobicity and water repellency of 100% cotton fabric were obtained by Ar plasma treatment followed by immersion in an ethanol solution of oleic acid [22] or by surface treatment with helium/1,3-butadiene plasma at atmospheric pressure [23]. 

Cellulose films or sheets have also been plasma treated for different purposes. For example, films of regenerated cellulose treated by low pressure oxygen plasma showed different surface chemistry and topography [24]. Similarly, dielectric barrier discharge (DBD) plasma treatment of a cellulose film at atmospheric pressure using N_2_/NH_3_ (90/10) gas mixture led to surface functionalization with amine and amide groups, which promoted cell differentiation [25]. Natural terpenes (limonene and myrcene) grafted on cellulose sheets using vacuum plasma modified the cellulose substrate from highly hydrophilic to hydrophobic [26] and fatty acids (butyric acid and oleic acid) grafted on unbleached and bleached kraft pulp using vacuum plasma increased the hydrophobicity of cellulose fibers [27]. Conversely, O_2_ plasma treatment of bacterial cellulose membranes led to a decrease of the effective pore area and water flux and to increased hydrophilicity, which are important for filtration [28]. On the other hand, lyophilized bacterial cellulose sheets treated with nitrogen plasma under vacuum showed increased porosity and enhanced adhesion of endothelial and neuroblast cells due to the functional nitrogen groups grafted on the surface of cellulose [29]. 

The application of DBD plasma for the surface modification of cellulose fibers or textiles has been extensively studied [19,30,31] but in the case of cellulose nanofibers/nanocrystals treated by DBD, the literature is extremely scarce. Only in a recent study [32], cellulose nanofibers from water suspension were deposited on glass and the resulted coatings were treated by DBD plasma in helium gas. The treatment increased the content of carbonyl and carboxyl groups on the nanocellulose coatings and their roughness. However, the plasma treated nanocellulose obtained by this method cannot be re-dispersed in water or other solvents due to hornification [33] and it cannot be used in dried form as a reinforcing agent, for enhancing the properties of polymers [10]. 

Submerged liquid plasma (SLP) is a recent hot topic and a valuable method to obtain or modify nanostructured materials [34,35]. So far, SLP has mainly been used as a low energy synthesis method to produce nanostructured carbon materials. Thus, nitrogen-functionalized graphene was produced by applying a high electric potential between graphite and Pt electrodes in acetonitrile solvent [35]. This product showed a good dispersibility in both hydrophilic and hydrophobic solvents. SLP treatments produce several active agents, highly reactive species, radicals, charged particles, ozone, ultraviolet radiation, and shockwaves in the aqueous suspension [36] which may lead to the surface functionalization of nanocellulose. It is expected that the application of cold plasma directly to the water suspension of cellulose nanofibers is much more suited to obtain cellulose nano-reinforcements with a modified surface for polymer composites. Besides the advantages of using plasma sources at atmospheric pressure (without vacuum components, no chamber limitation and easy operation at low cost), plasma treatments in liquid have the great advantage of enabling the treatment of nanoparticles or nanofibers as suspensions in liquids. 

In this work, the nanocellulose liquid suspensions were treated by using two types of non-thermal plasma sources developed for surface treatment, which are conceptually different: (i) a filamentary plasma jet based on dielectric barrier discharge (DBD) [37,38] and (ii) a plasma torch [39]. To the best of our knowledge, this is the first example of the use of SLP for the surface treatment of nanocellulose and the first time a filamentary jet discharge with dielectric barrier is used submerged in liquid. DBD filamentary jets were previously used for polymer deposition [40] but not immersed in the liquid where the material to be treated is dispersed. Both sources can be operated at low radiofrequency power (around 100 W) in continuous Ar flow. These atmospheric pressure plasma sources were previously used for the surface treatment of polymers [41], decomposition of dye solutions [42] or functionalization of graphene suspensions [43]. These plasma sources work properly both in open atmosphere and completely immersed in various liquid media, such as nanocellulose water suspensions or graphene suspensions. The aim of this work was to investigate the effect of these plasma sources in different operating conditions on the surface properties of nanocellulose (NC) obtained by mechanical disintegration of BC membranes and designed as reinforcing agent in biopolymers. NC surface changes were examined by Fourier transform infrared spectroscopy (FTIR), X-ray photoelectron spectroscopy (XPS), and thermogravimetric analysis (TGA). Plasma treated NC samples were tested as reinforcing agents in a biopolymer matrix, poly (3-hydroxybutyrate) (PHB). 

## 2. Materials and Methods

### 2.1. Materials

Bacterial strain Gluconacetobacter Xylinus DSM 2004, purchased from Leibniz Institute DSMZ (German Collection of Microorganisms and Cell Cultures, Braunschweig, Germany), was used for the biosynthesis of bacterial cellulose (BC) membranes. Acetonitrile 99% (ACN) was purchased from Fluka (Buchs, Switzerland) and used as received. PHB type P304 with a tensile strength of 28 MPa (ISO 527, 50 mm/min) and a Charpy impact strength of 60 kJ/m^2^ (ISO 179, 23 °C) was purchased from Biomer (Krailling, Germany). All the other chemicals were of analytical grade and were purchased from Sigma-Aldrich (St. Louis, MI, USA).

### 2.2. Production of BC Membranes; Defibrillation of BC Membranes to Obtain Nanocellulose 

**Figure 1:**
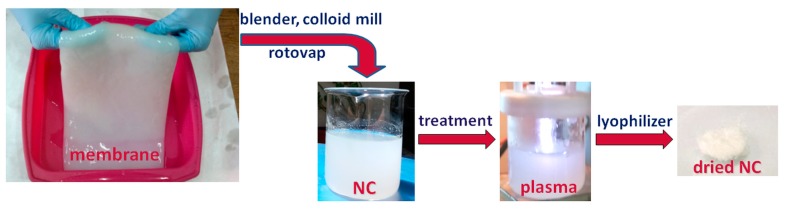
Schematic representation of defibrillation and plasma treatment of nanocellulose.

The culture medium used for the fermentation of Gluconacetobacter Xylinus contained 7.5% glucose equivalents from poor quality apples extract, 2% glycerol, 0.2% ammonium sulfate, and 0.5% citric acid. This culture media (50 mL) was autoclaved at 121 °C, for 15 min and, after cooling, it was inoculated with 10% (*v*/*v*) stock culture (Gluconacetobacter Xylinus on Schramm–Hestrin medium). BC membranes were produced under static conditions at 30 °C for 14 days. After the treatment with sodium hydroxide and sodium azide for cell lysis and reduction of microbial contamination, the BC membranes were neutralized with 1% acetic acid and washed several times with distilled water. NC was obtained from fresh BC membranes by mechanical defibrillation (Figure 1) using a blender and a colloid mill, as described in [10]. 

The diluted suspension of NC in water was concentrated using a rotary evaporator (Heidolph, Schwabach, Germany) and then plasma treated. Following this step, different treated NC suspensions were lyophilized using a FreeZone 2.5 L (Labconco, Kansas City, MO, USA) resulting in dried surface-treated NC. 

### 2.3. Plasma Treatment of NC

**Figure 2:**
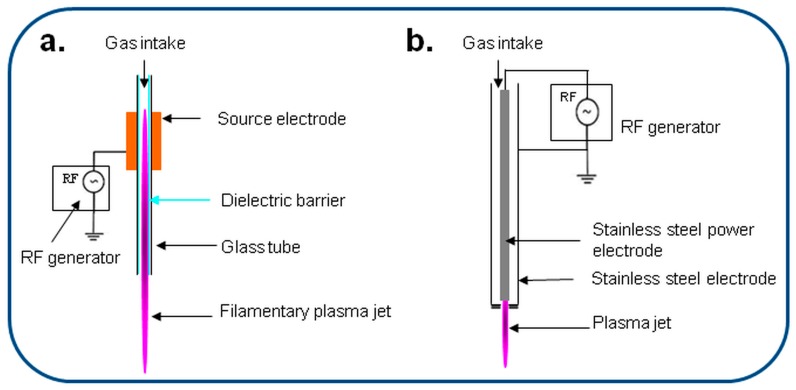
Configuration of plasma jet sources: dielectric barrier discharge (DBD) (**a**) and plasma torch (E) (**b**).

The two types of plasma sources that were used for the surface functionalization of NC are shown in Figure 2. The DBD plasma source with floating electrode is shown in Figure 2a whilst the plasma torch (E) with the expanding plasma jet in contact with electrodes is presented in Figure 2b. Both sources are capacitively coupled with a radiofrequency (RF, 13.56 MHz) power supply and may produce an external jet of over 40 mm, depending on the operation parameters (gas flow, RF power). Both discharges are initiated in open atmosphere and, then, the jets are completely immersed in the water suspension for the surface functionalization of NC. The conditions used for the plasma treatments are shown in Table 1. Both plasma sources were designed for atmospheric pressure [39,40] and the plasma torch was previously tested completely immersed in liquid for the functionalization of graphene [43]. 

A typical experiment for the surface treatment of NC consists of the ignition of a plasma operating at atmospheric pressure in a 3000 sccm argon (Ar) flow and a power of 100 W, injecting the reactive gases (oxygen, nitrogen or ammonia) and immersing the plasma jet in the liquid suspension (50 mL) for 15–30 min. The concentration of NC in the liquid suspension was 1 wt %. Acetonitrile was also introduced into the water suspension of NC, the rest of the process parameters being identical. Droplets from the plasma treated NC suspensions were dried on silicon substrates for further investigations (XPS, FTIR). Plasma treated NC suspensions were lyophilized to obtain dried NC with a modified surface. Due to the characteristics of the second plasma source type, more nitrogen was used for the surface treatment of NC and a higher RF power (Table 1, E15 and E30). Dried surface-treated NC samples were used in small concentration (0.2 wt %) as reinforcement in PHB. The nanocomposites were obtained in the mixing chamber of a Brabender LabStation (Duisburg, Germany) at 165 °C for 10 min, rotor speed of 50 min^−1^. For mechanical characterization, films with the thickness of about 200 µm were obtained by compression molding in an electrically heated press (Dr. Collin, Ebersberg, Germany) at 175 °C, with 120 s of preheating (5 bar) and 75 s under pressure (100 bar). The films were quickly cooled down in a cooling cassette. 

### 2.4. Characterization

Scanning electron microscopy (SEM) images of NC were obtained using a Quanta Inspect F scanning electron microscope (FEI-Philips, Hillsboro, OR, USA)) with a field emission gun at an accelerating voltage of 30 kV with a resolution of 1.2 nm. NC samples were directly mounted on the adhesive tape and sputter-coated with gold for 30 s before examination. Plasma treated NC films deposited on silicon substrates were examined by atomic force microscopy (AFM) using a Bruker MultiMode 8 (Santa Barbara, CA, USA) in Peak Force QNM (Quantitative Nanomechanical Mapping) mode. The images were captured using a silicon tip with the spring constant of 40 N/m and a resonant frequency of 300 kHz at a scanning rate of 0.9 Hz. Tensor 37 spectrometer with attenuated total reflectance (ATR) setup from Bruker Optics (Ettlingen, Germany) was used to examine the surface chemistry changes and to record the Fourier transform infrared spectroscopy (FTIR) spectra of the NC films deposited on silicon substrates. The spectra were collected in duplicate, at room temperature, from 4000 to 400 cm^−1^. All the spectra were the average of 16 scans at a spectral resolution of 4 cm^–1^. The NC treated under different experimental conditions, deposited on silicon substrates was also analyzed by X-ray photoelectron spectroscopy (XPS) using a ESCALAB™ XI+ spectrometer (Thermo Scientific, Waltham, MA, USA) with a monochromatic Al Kα source at 1486.6 eV. The XPS spectra were recorded as survey spectra with the pass energy of 100 eV (for 10 scans) and the high-resolution scans in the C1s, O1s and N1s regions, with the pass energy of 20 eV and resolution 0.1 eV (for 20 scans). Thermogravimetric analysis (TGA) of both plasma treated NC and nanocomposites was performed on a TA-Q5000 V3.13 (TA Instruments Inc., New Castle, DE, USA) using nitrogen as the purge gas at a flow rate of 40 mL/min. A heating cycle from 25 °C to 700 °C at a heating rate of 10 °C/min was applied. The experimental error was less than ±0.5 °C for all the characteristic temperatures. Tensile properties of PHB/NC nanocomposites were measured according to ISO 527, at room temperature, on five specimens for each nanocomposite, using an Instron 3382 universal testing machine (Instron Corporation, Norwood, MA, USA) and a crosshead speed of 2 mm/min.

## 3. Results and Discussion

### 3.1. NC Morphology before and after Plasma Treatments

**Figure 3:**
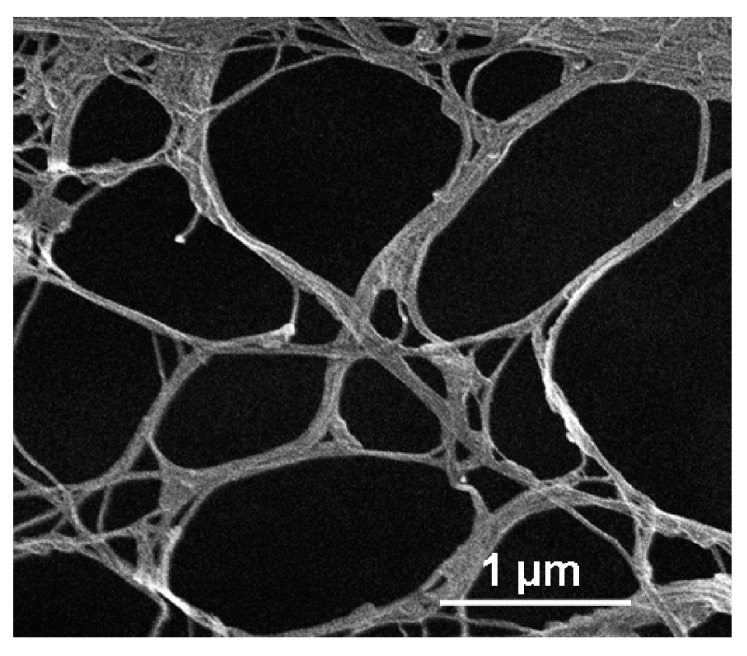
Scanning electron microscopy (SEM) image of nanocellulose (NC) from defibrillated membranes showing a sparse network of nanofibers.

The nanometric dimensions of cellulose fibers obtained by the disintegration of BC pellicles provide a high surface area for plasma treatment. Indeed, in the SEM image of dried NC (Figure 3) it is shown that BC pellicles were disintegrated resulting in a sparse network of cellulose nanofibers with thickness between 40 and 100 nm. 

**Figure 4:**
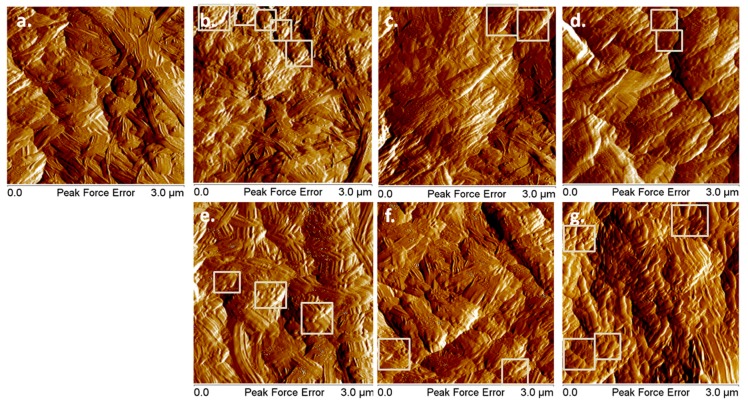
Atomic force microscopy (AFM) images (peakforce error) of untreated (**a**) and plasma treated NC: Ar (**b**); Ar/O_2_ (**c**); Ar/N_2_ (**d**); Ar/N_2_ (E30) (**e**); Ar/NH_3_ (**f**); Ar-ACN (**g**); Regions of interest showing small-length nanofibers agglomerations are framed in squares.

NC films deposited on silicon substrates were examined by AFM before and after the plasma treatments (Figure 4). 

Both individual nanofibers and fibers bundles were noticed in the AFM images on the surface of all the films, regardless of the treatment, but with different surface morphologies and arrangement of the nanofibers. For example, more small-length nanofibers were observed after plasma treatments which allow a more dense arrangement. These nanofibers were noticed as heaps containing agglomerations of small fibers on the surface of the films and they were framed with squares in Figure 4. More such regions of interest were noted on the surface of Ar treated NC and NC Ar-ACN samples, suggesting a more efficient treatment. This may be due to the plasma treatments, which led to the detachment of nanofibers from the network and their breaking. Similarly, mechanical treatments, acid hydrolysis or ultrasonic treatments led to the decrease in cellulose fiber size [44,45].

**Figure 5:**
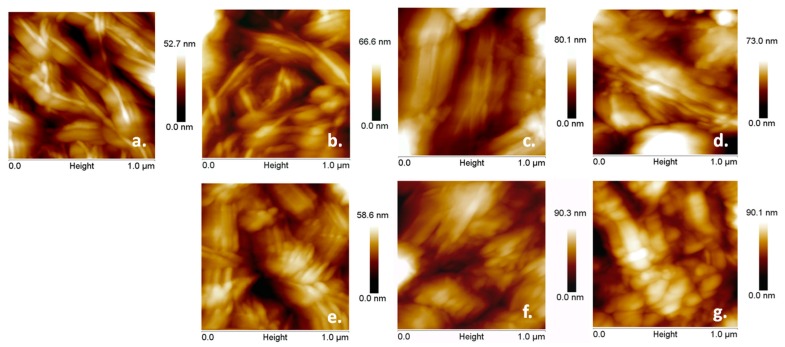
AFM topographic images of untreated (**a**) and plasma treated NC: Ar (**b**); Ar/O_2_ (**c**); Ar/N_2_ (**d**); Ar/N_2_ (E30) (**e**); Ar/NH_3_ (**f**); Ar-ACN (**g**).

More information can be extracted from the higher magnification AFM images shown in Figure 5. Before plasma treatment, the NC mostly appears as individual tapes longer than 1 µm, and with a smooth surface. After Ar treatment, both individual twisted tapes and small size fragmented fibers were noticed. Such fragments, similar to nanoparticles, were observed in the bottom right corner of Figure 5b. Ar/O_2_, Ar/N_2_, Ar/N_2_ (E30), and Ar/NH_3_ treatments led to more agglomerated and fragmented NC tapes compared to Ar treatment. The smallest fibers in this series were observed after DBD plasma treatment with Ar/NH_3_ and torch plasma in Ar/N_2_ (Figure 5e,f). However, Ar-ACN treatment was the most efficient in lowering the size of NC and mostly NC particles and fragmented tapes were observed on the surface of this film (Figure 5g). 

For a quantitative analysis, the root mean square roughness (R*_RMS_*) of the NC films surface was measured with the NanoScope software. Three different locations of square shape (3 × 3 μm) were analyzed for each sample and the R*_RMS_* was an average of these values. The R*_RMS_* of the NC Ar/N2 (E30) and NC Ar surfaces was 58 ± 3 nm and 48 ± 4 nm, respectively, lower than that of the other films which varied from 70 ± 3 (for the reference) to 78 ± 4 for NC Ar/N_2_ and NC Ar/O_2_. This suggests a more compact arrangement of the fibers on the surface of the first samples. 

Morphological investigation on the surface of plasma treated NC deposited as films on silicon substrates emphasize the different effects of plasma treatments depending on the type of the source (DBD plasma jet or torch), the reactivity of the gas (Ar, N_2_, O_2_, NH_3_) or the liquid (water or ACN). 

### 3.2. Thermal Analysis of NC before and after the Treatments

**Figure 6:**
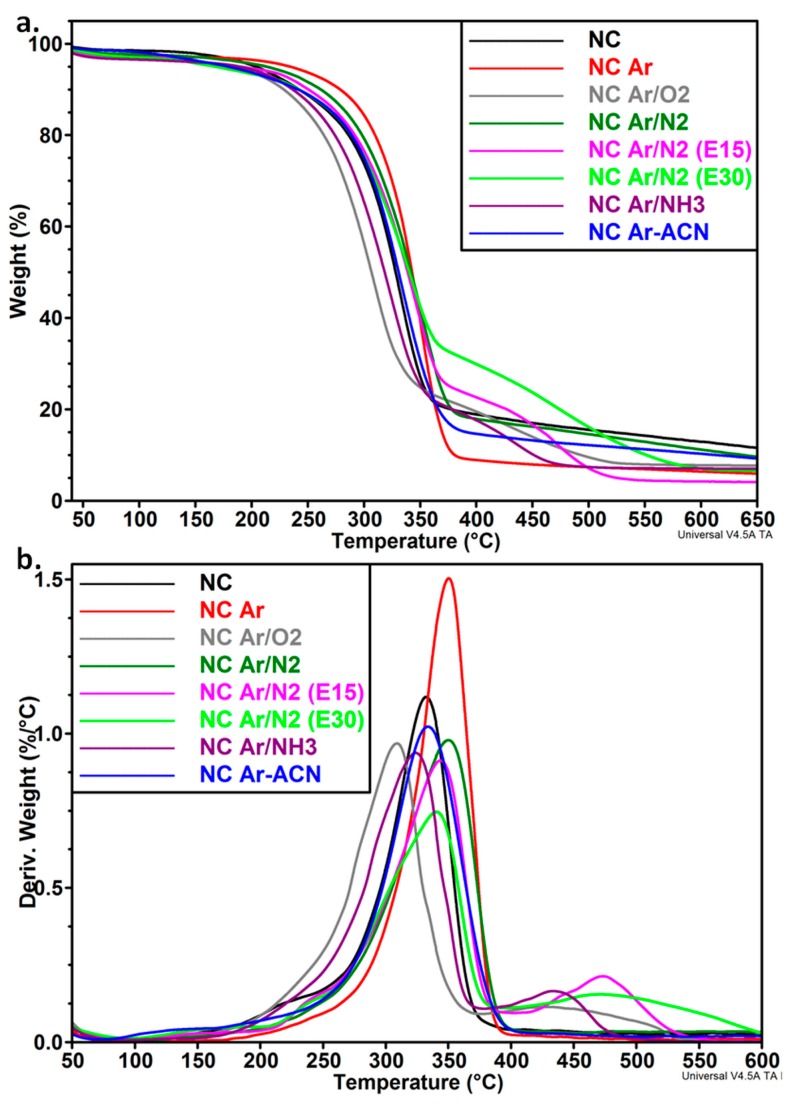
Thermogravimetric analysis (TGA) spectra (**a**) and derivative thermogravimetric (DTG) overlapped curves (**b**) of plasma treated NC.

Thermal analysis may give valuable information on the structural changes of NC after plasma treatments due to the fact that these changes are reflected in a different thermal behavior. The different conditions of plasma treatment influenced the thermal behavior of NC as shown in Figure 6a,b. The temperature at the maximum degradation rate (*T_max_*), the onset degradation temperature (*T_on_*), and the temperature at 10% weight loss (*T*_10%_) as well as the residue at 700 °C (*R*) and the residue after the first decomposition step (*R_I_*) are summarized in Table 2. 

Pristine NC and DBD plasma treated NC with Ar (in water or water–ACN) and with Ar/N_2_ gases showed only a one-step degradation process and a *T_max_* at 330–350 °C. This peak (Figure 6b) is commonly attributed to the dehydration and depolymerization of the cyclic structures of cellulose [46]. DBD plasma treatment using more reactive gases (O_2_ or NH_3_ in Ar) and the treatment in Ar/N_2_ mixture using plasma torch and harsher conditions, characteristic of this plasma source, led to a two-step degradation pattern. The different degradation processes highlighted by TGA correspond to different structures. A good correlation between TGA results and cross-linking was previously reported [47]. Cellulose cross-linked with epichlorohydrin at variable levels showed the main decomposition events at different temperatures depended on the cross-linking degree, the higher temperature events being attributed to covalent bond cleavage of the polymer network. Thus, it may be presumed that the second degradation process around 437 °C in the case of NC Ar/O_2_ and NC Ar/NH_3_ or around 475 °C and 486 °C, for NC Ar/N_2_ (E15) and NC Ar/N_2_ (E30), respectively, may be assigned to the cross-linked cellulose. Hydroxyl radicals, electrons, and ions were formed during these treatments which may break the cyclic structures and favor the cross-linking reactions. 

The concentration of the cross-linked polymer may be roughly estimated from the residue after the first decomposition step, 20%–23% for 30 min of DBD plasma exposure and 24% or 32% when plasma torch was used for 15 or 30 min. This means that the changes induced by the treatments depend both on the type of the source and time of exposure. A small shoulder occurred only for unmodified NC, at about 225 °C; it is usually associated with the release of water or other low molecular weight fractions from defibrillated BC [48]. No shoulder was detected in this temperature range for NC after plasma treatment. This may lead to the conclusion that the treatment with filamentary plasma jet and plasma torch in Ar or gas mixtures was able to remove the bond water and low molecular weight impurities from NC. In addition to removing water, the plasma torch treatment may cause some chemical changes which led to the release of a small concentration (6%–7%) of low molecular weight fractions around 240 °C. 

The DBD plasma treatment in Ar and Ar/N_2_ mixture significantly increased the *T_on_* value of NC, with 35 °C and 17 °C, respectively. An increase of the characteristic temperatures was also observed in the case of plasma torch for NC Ar/N_2_ (E15) (Table 2). The higher thermal stability may be caused by the removal of less thermally stable components, including low molecular weight fractions and impurities. The release of water and slight cross-linking may also be caused by the milder conditions of the filamentary plasma treatment and the plasma torch for small duration. A slight increase of *T_on_* with 3 °C was reported for Quiscal fibers after atmospheric DBD plasma treatment [31]. An increase of the decomposition temperature with about 20 °C was also observed for jute fibers treated with Ar plasma under vacuum [49]. However, natural fibers like Quiscal or jute fibers contain hemicelluloses and lignin in a high proportion which greatly influence the thermal stability during the plasma treatment. Generally, the literature regarding the influence of plasma treatment on the thermal stability of cellulose is rather scarce and no information regarding nanocellulose is available. 

No significant change in *T_on_* and *T_max_* values was noticed for NC Ar-ACN and NC Ar/N_2_ (E30) plasma treatments compared to untreated nanocellulose, however a decrease of the characteristic temperatures was obvious for the plasma treatment with more reactive gases, O_2_ or NH_3_ in Ar (Table 2). Although used in small amount in the Ar flow, NH_3_ and, especially, O_2_ decrease the *T_on_* with 16 °C and 24 °C, respectively. A slight decrease of the thermal stability was observed for vacuum plasma treated Arundo fibers exposed to a plasma power of 150 W for 120 s [50]. In contrast, a decrease of the final degradation temperature with 107 °C was observed for air plasma treated gray linen compared to the untreated sample [51]. Again, the non-cellulosic components of natural fibers have definitely influenced the thermal stability. It is worth mentioning that all the treatments led to a lower residue, which highlights the efficiency of these treatments in cleaning the nanocellulose from low molecular weight impurities. 

### 3.3. Surface Analysis by ATR-FTIR

**Figure 7:**
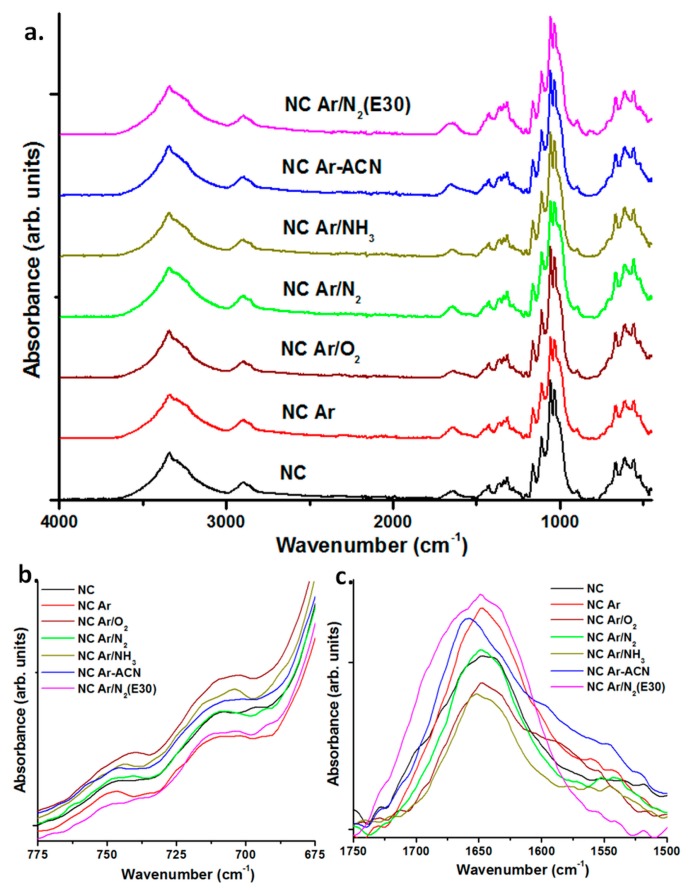
Fourier transform infrared spectroscopy (FTIR) spectra of untreated and plasma treated NC (**a**); zoomed-in regions (775–675 cm^−1^) (**b**) and (1750–1500 cm^−1^) (**c**) of the same spectra.

The FTIR spectra of NC before and after plasma treatments are shown in Figure 7a. For comparison, all FTIR spectra were normalized using the C−H stretching vibration from 2897 cm^−1^ [52]. Regarding the crystalline structure, NC is a mixture of Iα and Iβ allomorphs with Iα the predominant crystal form [10] since it was obtained from BC by mechanical disintegration. The presence of the two allomorphs was detected in all the samples, untreated and plasma treated, at 750 cm^−1^ (assigned to the contribution of cellulose Iα) and at 710 cm^−1^ (corresponding to cellulose Iβ) [10,53]. No significant change in the intensity ratio and position of the bands corresponding to the two allomorphs was observed after the plasma treatments, regardless of the source or conditions (Figure 5b); this shows that the crystalline phase in NC is resistant to the plasma attack. 

The O–H groups in cellulose are hydrogen bonded and the overlap of several O–H stretching modes leads to a broad peak as that observed in Figure 7, between 3000 and 3600 cm^−1^. The most important vibrations were noticed at about 3242 cm^−1^ for cellulose Iα and were assigned to intra-chain hydrogen-bonded 2OH [54] and between 3300 and 3350 cm^−1^ for coupled vibrations corresponding to inter and intra-chain hydrogen bonded 2OH, 3OH, and 6OH groups [54]. The OH peak position is shifted to a lower wavenumber because of hydrogen bonding [54,55]. The assignment of OH absorption bands is complicated by the contribution from N–H stretching vibrations in amide groups, knowing that BC may contain a small amount of proteins. The main peak shifted from about 3340 cm^−1^ for NC and NC Ar, to 3343 cm^−1^ for NC Ar/O_2_ and NC Ar-ACN, to 3342 cm^−1^ for NC Ar/N_2_ and NC Ar/NH_3_ and to 3341 cm^−1^ for NC Ar/N_2_ (E30). This slight shift to higher wavenumber is an indication of the decrease of hydrogen bonding due to cross-linking or participation of OH in other bonds [54,56] and, possibly, due to the presence of N–H vibration from newly created bonds [57]. 

The FTIR spectra in the range from 1500 to 1750 cm^−1^ show important differences (Figure 7c). This is a region where several vibrations overlap. For example, H–O–H bending absorption [58] and C=O stretching vibration in amide I [59] are assigned at 1635–1650 cm^−1^, N–H and C–N vibrations in amide II at about 1550 cm^−1^ [59,60], aliphatic and aromatic C=C stretching and C=N stretching vibrations in the range 1600–1660 cm^−1^ [61,62,63]. It is worth mentioning that the peak at 1647–1648 cm^−1^ in pristine NC and plasma treated NC with Ar, Ar/N_2_ and Ar/O_2_ is shifted to a higher wavenumber in the case of Ar/NH_3_ (1651 cm^−1^) and Ar-ACN (1658 cm^−1^) treatments. This may be due to the formation of new C=O or C=C bonds in the case of the last two treatments. In addition to the peak at 1648 cm^−1^, the NC treated with plasma torch (E30) shows a wide shoulder stretching from 1660 to 1700 cm^−1^, with a local peak at about 1665 cm^−1^. This shoulder may be assigned to C=O stretching vibration and C=N stretching in imines and oximes [63]. 

The shoulder at 1550 cm^−1^ which is generally associated with N–H and C–N vibrations in amide II [59,60] is hardly visible for pristine NC, because of the low content in amide from the protein residues of the bacterial cells, but in the case of Ar/N_2_ and Ar/NH_3_ treatments this is obvious. Likewise, a wide shoulder is observed in the range from 1500 to 1600 cm^−1^ in the case of Ar, Ar/O_2_ and, especially, Ar-ACN plasma treated samples (Figure 7c). Considering the shift of the main peak and the new shoulders which appear after plasma treatments it may be concluded that both DBD and torch plasma sources led to nitrogen containing groups on the surface of NC. However, the assignment of vibration modes is difficult because of the overlapping of vibrations and XPS analysis was undertaken to obtain more information on the type of nitrogen containing bonds on the surface of cellulose. 

### 3.4. Surface Analysis by XPS 

The surface chemistry of pristine NC and submerged plasma treated samples were investigated by XPS. Carbon, oxygen, and small amount of nitrogen were identified in the general XPS spectra. The relative atomic concentration of elements and oxygen/carbon and nitrogen/carbon ratio are presented in Table 3.

From the survey spectra, one can see the tendency of NC oxidation after plasma treatment. Regarding oxidation efficiency, DBD filamentary jet in pure Ar is more efficient (O/C = 0.57) compared with similar treatments performed in Ar/O_2_, Ar/N_2,_ and Ar/NH_3_ mixtures (O/C lower than 0.55), but DBE treatments produce by far the highest oxidation; the O/C ratio increased from an initial 0.55 value to 0.61 after 15 min and 0.63 after 30 min of DBE treatment. Concerning N incorporation, the highest N/C ratio was observed for the Ar-ACN treatment with the DBD source.

The presence of nitrogen in untreated NC comes from the biosynthesis and is caused by the proteins from cell debris. Slight change of N 1s% between samples was observed, with a clear increase of nitrogen content in the case of filamentary plasma in Ar submerged in ACN. 

**Figure 8:**
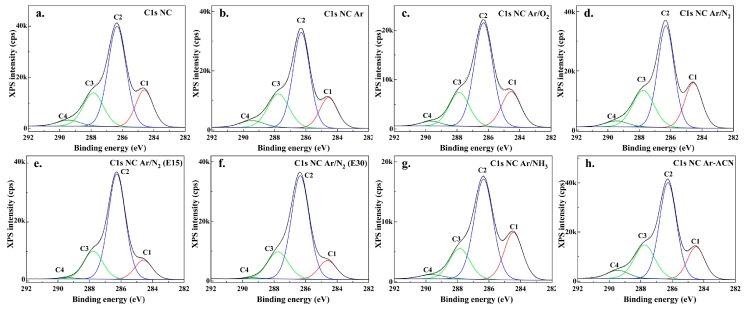
High resolution spectra of C1s region with deconvolution (different colored C1–C4 components) for pristine NC (**a**) and plasma treated NC: NC-Ar (**b**); NC Ar/O_2_ (**c**); NC Ar/N_2_ (**d**); NC Ar/N_2_ (E15) (**e**); NC Ar/N_2_ (E30) (**f**); NC Ar/NH_3_ (**g**), and NC Ar-ACN (**h**).

In order to evaluate the elemental bonding states at the samples surface, high resolution spectra were measured for C1s, O1s, and N1s regions. Each C1s spectrum (Figure 8) was deconvoluted in four components which were assigned according to the binding energy to: (i) C–C and C–H bonds for C1s at about 284.6 ± 0.1 eV (C1), (ii) single bonded C in C–O (ether, hydroxyl) or C–N at 286.3 ± 0.1 eV (C2), (iii) double bonded C in C=O (carbonyl) or O–C–O (acetal) at about 287.9 ± 0.1 eV (C3), O–C=O (carboxyl or ester) at 289.5 ± 0.1 eV (C4). Similarly, the O1s regions contain three sub-peaks centered on the binding energy of 531.0 ± 0.1 eV (O1: O=C in carbonyl and ketone), 532.9 ± 0.1 eV (O2: single bonded O, in hydroxyl or epoxy), and 534.9 ± 0.1 eV (O3: attributed to carboxyl or ester groups). The percentage of each component, with respect to the C1s and O1s peak areas, are presented in Table 4.

In the C1s region, the predominant peaks correspond to C–O (C2 sub-peak). The percentage of C2 components varies between 49.4% and 66.9%, depending on the treatment. From the high-resolution spectra in the carbon region one can observe that all the treatments induced changes and reorganization of chemical groups on the NC surface. The filamentary DBD treatments mostly led to diminished C2 component, while plasma torch to increased C2 component with respect to pristine NC. 

Regarding the C1 component, i.e., assigned to low molecular weight fractions and impurities, one can observe a reduction of C–C/C–H bonds (cleaning of NC) by the Ar plasma and, especially, torch treatments. In addition to NC cleaning process, torch treatments (E15 and 30 samples) also led to the decrease of C3 and C4 components and, therefore, the increase of C–O bonds at the expense of C–C/C–H (C1) and O–C=O (C4) bonds was noticed. It is likely that the contaminant carbon, rich in alkyl and carboxyl groups, was removed by plasma torch treatments. Still, an increase of the C4 component was observed in the case of Ar and Ar-ACN DBD treatments, possibly due to a higher affinity to water molecules and oxidation. This fact was also confirmed in the O1s region. 

Regarding the concentration of O1, O2, and O3 components (see Table 4) one can observe that the majority of oxygen (O2 peak) is distributed in relation to C2 component and only few percentages in combination to C1 and C3. The results confirm the purification of NC after torch treatments where O2 component reaches a maximum of 96.1%–96.4%. 

**Figure 9:**
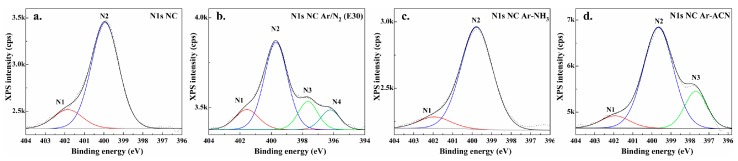
High resolution N1s spectra with deconvolution (different colored N1–N4 components) of pristine NC (**a**); NC Ar/N_2_ (E30) (**b**); NC Ar/NH_3_ (**c**), and NC Ar-ACN sample (**d**).

From the N1s region (Figure 9), it is seen that the untreated NC presents two types of nitrogen bonds, with binding energies at 401.8 eV (N1) and 399.9 eV (N2). 

The N1 (15.8%) and N2 (84.2%) peaks on NC are associated to: protonated amines or lactam [64] and to amides, alkylamides or amines, respectively [65,66]. We observed that all treated samples keep the native nitrogen, with a slight variation of the total percentage. In addition, the introduction of new nitrogen bond types was noticed. Compared to pristine NC, the torch treatment in N_2_ for 30 min and the DBD in NH_3_ and ACN produced the largest changes. New peaks occurred after these treatments leading to changes of the percentage of bonds; the treatment in acetonitrile modified the percentage of N1 (8.4%) and N2 (71.6%) components, by introducing new nitrogen containing bonds at about 398 eV, with a relative concentration of 20%, assigned to the iminic group [67] (component N3). Another additional peak was observed after nitrogen treatment by torch (E30), at around 396.5 eV, assigned to the pyridinic C=N [68] (component N4). The concentration of N1, N2, N3, and N4 components for samples (E30) were 11%, 16.1%, 59.2%, and 13.7%, respectively. Ammonia plasma even increase the content of amine groups N2 (89.8%) at the expense of N1 component (10.2%) with respect to untreated NC. Therefore, the source type and the gas mixture as well as the liquid medium influenced the functionalization of the nanocellulose surface. 

### 3.5. Effect of Plasma Treated NC on PHB Properties

**Figure 10:**
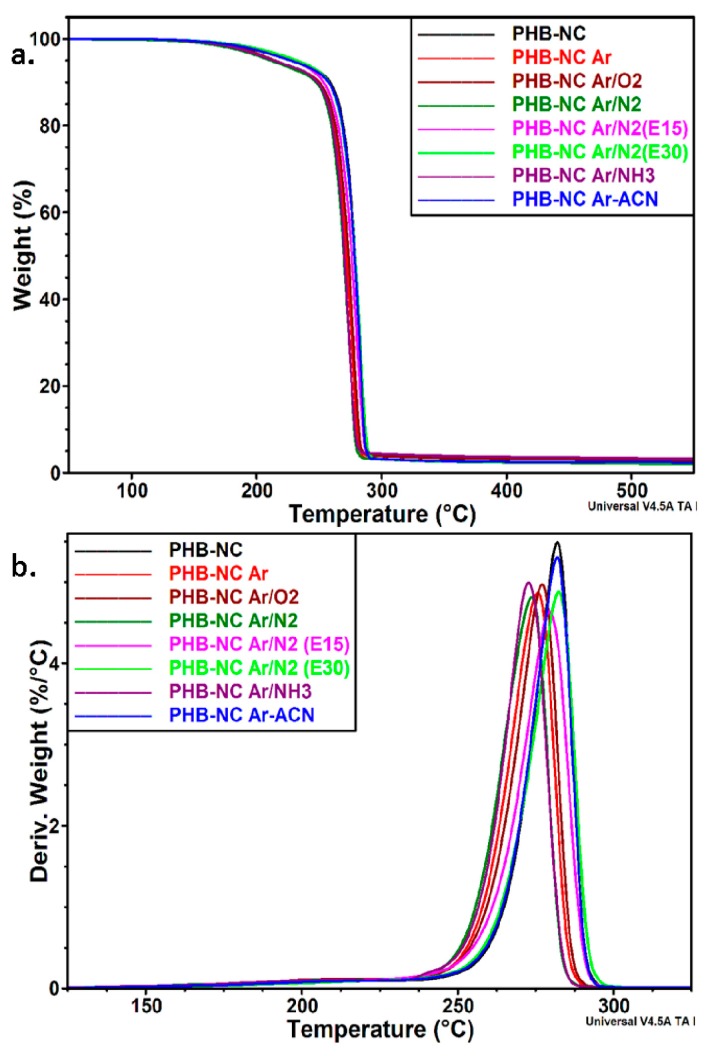
TGA (**a**) and DTG (**b**) curves for poly (3-hydroxybutyrate) (PHB) nanocomposites containing plasma functionalized NC.

The analysis of submerged liquid plasma treated NC samples by FTIR, XPS, and TGA showed that nanocellulose was surface functionalized by oxidation and bonding of nitrogen containing groups. The effect of these functionalized NC samples on the thermal stability and mechanical properties of a biopolymer with high expectations for biomedicine (i.e., PHB) was also studied. To differentiate the effect of each NC surface modification, a low concentration (0.2 wt %) of submerged liquid plasma functionalized NC was used in the melt compounded PHB nanocomposites. The thermal degradation of PHB nanocomposite containing pristine NC is a two-step process (Figure 10a,b), with a small shoulder at 215–220 °C, characteristic to the decomposition of NC and a main degradation step at about 282 °C, characteristic of PHB. 

PHB containing plasma functionalized NC showed similar thermal behavior compared to that of PHB-NC. Similar *T_on_* and *T_max_* values for the nanocomposites with NC, NC Ar/N_2_ (E30) and NC Ar-ACN are shown in Table 5. Slightly lower values were obtained for the rest of the samples. However, the shift of the characteristic temperatures was less than 9 °C. The efficiency of a reinforcing fiber is decisively influenced by the fiber–polymer interface, a good interface adhesion leading to a better stress transfer from the polymer to the fiber and to a higher strength [69]. The mechanical properties of PHB nanocomposites were measured in tensile mode (Table 5). Although used in a small amount in PHB, plasma functionalized NC induced an increase of the tensile strength, with 10%–15%, compared to the nanocomposite with the same amount of pristine NC. This shows the enhancement of the adhesion between PHB and surface treated NC probably because of the new functionalities on the fiber surface induced by plasma treatments and emphasized by FTIR and XPS. 

The Young’s modulus is measured using the stress and strain values in the elastic region, where the deformation is small and, therefore, it is less influenced by the adhesion at the fiber– polymer interface [70]. The values of Young’s modulus in Table 5 also show smaller influence of the different treatments compared to the tensile strength. The cumulative analysis of thermal and mechanical behaviors shows that Ar treatment of NC in ACN-water suspension has the best influence on the properties of PHB, an increase of the tensile strength with 18%, a slight increase (6%) of Young’s modulus and good thermal stability, similar to that of PHB and PHB-NC. It is worth mentioning that there is a good correlation between these results and the structural analyses (FTIR and XPS) and morphological investigation of the Ar-ACN treated NC. 

## 4. Conclusions

This study demonstrated that the application of a plasma torch and filamentary jet plasma modified the morphology and surface chemistry of nanocellulose fibers dispersed in a liquid phase. The plasmas were operated in Ar or mixtures of Ar with O_2_, N_2_, NH_3_, while the liquid was water or ACN in water. The treatment with filamentary plasma jet and plasma torch in Ar or gas mixtures was able to remove the bond water and low molecular weight impurities from NC. All the treatments led to a lower residue, which highlights the efficiency of these treatments in cleaning the nanocellulose. Individual small length nanofibers in higher proportion were formed after the plasma treatments which may favor a more homogeneous dispersion in nanocomposites. However, the intensity of this effect was different depending on the type of the source and gas mixture. FTIR and XPS measurements showed that both oxygen and nitrogen functional groups were formed on the surface of NC depending upon conditions. The functionalization with oxygen moieties was more effective in the case of the plasma torch system in water and the functionalization with nitrogen moieties was most effective using the filamentary jet in ACN media. Both functionalization types can promote the adhesion of components in nanocomposites. Indeed, small proportion of plasma functionalized NC using the filamentary jet led to 10%–15% increase of the tensile strength, compared to the nanocomposite with the same amount of untreated NC. This work provides a new eco-friendly method for the surface functionalization of nanocellulose directly in its water suspension which overcomes the disadvantages of polluting, time consuming, and complex chemical treatments.

## Figures and Tables

**Table 1:** Conditions for dielectric barrier discharge (DBD) and plasma torch (E) plasma treatments of nanocellulose (NC).

Samples	Ar flow (sccm)	Reactive Gas/Liquid	RF Power (W)	Treatment Time (min)
NC Ar	3000	-	100	30
NC Ar/O_2_	3000	O_2_ (5 sccm)	100	30
NC Ar/N_2_	3000	N_2_ (10 sccm)	100	30
NC Ar/N_2_ (E15)	2000	N_2_ (1500 sccm)	250	15
NC Ar/N_2_ (E30)	2000	N_2_ (1500 sccm)	250	30
NC Ar/NH_3_	3000	NH_3_ (5 sccm)	100	30
NC Ar-ACN	3000	ACN 30% in water	100	30

**Table 2:** Thermogravimetric analysis (TGA) results for plasma treated NC.

Samples	*T*_10%_ (°C)	*T_on_*(°C)	*T_max_*(°C)	*R_I_*(%)	*T’_max_*(°C)	*R *(%)
NC	244.0	284.1	332.1	-	-	10.6
NC Ar	278.6	310.2	350.3	-	-	5.5
NC Ar/O_2_	228.5	261.0	309.0	22.5	436.9	7.6
NC Ar/N_2_	261.4	294.3	350.1	-	-	8.1
NC Ar/N_2_ (E15)	250.4	290.2	343.6	23.8	474.5	4.1
NC Ar/N_2_ (E30)	241.9	282.6	340.2	31.8	486.4	6.5
NC Ar/NH_3_	237.3	268.4	323.6	20.4	436.6	6.9
NC Ar-ACN	241.2	289.0	333.8	-	-	8.3

**Table 3:** Relative atomic concentrations of carbon, oxygen, and nitrogen.

Samples	C1s (%)	O1s (%)	N1s (%)	O/C	N/C
**NC**	63.8	35.0	1.2	0.55	0.02
**NC Ar**	62.9	36.2	0.9	0.57	0.01
**NC Ar/O2**	63.5	35.1	1.4	0.55	0.02
**NC Ar/N2**	64.6	33.9	1.5	0.52	0.02
**NC Ar/N2 (E15)**	61.4	37.5	1.1	0.61	0.02
**NC Ar/N2 (E30)**	61.0	38.2	0.8	0.63	0.01
**NC Ar/NH3**	65.4	33.2	1.4	0.51	0.02
**NC Ar-ACN**	63.2	34.4	2.4	0.54	0.04

**Table 4:** Percentage of components from the total amount of carbon C1s and O1s.

Samples	C1s Components				O1s Components		
	C1 (%)	C2 (%)	C3 (%)	C4 (%)	O1 (%)	O2 (%)	O3 (%)
**NC **	19.7	55.3	20.7	4.2	2.9	87.5	9.6
**NC Ar**	17.9	53.2	23.5	5.4	3.1	85.2	11.7
**NC Ar/O2**	20.2	56.8	20.1	2.9	3.1	88.9	8.1
**NC Ar/N2**	23.1	49.4	23.6	4.0	4.0	85.0	11.0
**NC Ar/N2 (E15)**	12.5	66.9	19.7	0.8	2.2	96.1	1.7
**NC Ar/N2 (E30)**	12.1	66.9	20.3	0.7	1.9	96.4	1.8
**NC Ar/NH3**	24.9	54.5	17.6	2.9	3.6	88.0	8.4
**NC Ar-ACN**	18.3	53.6	22.6	5.6	3.4	82.7	13.9

**Table 5:** Thermal (*T_on_* and *T_max_*) and mechanical characteristics (tensile strength and Young’s modulus) of PHB-NC nanocomposites.

Nanocomposites	Tensile Strength (MPa)	Young’s Modulus (MPa)	*T_on_*(°C)	*T_max_*(°C)
**PHB-NC **	26.0 ± 1.4	1324 ± 62	269.0	281.9
**PHB-NC Ar**	29.5 ± 0.7	1358 ± 49	262.2	276.0
**PHB-NC Ar/O2**	29.0 ± 1.1	1387 ± 59	263.6	277.2
**PHB-NC Ar/N2**	28.5 ± 0.6	1395 ± 30	260.2	273.8
**PHB-NC Ar/N2 (E15)**	29.1 ± 0.5	1307 ± 41	265.5	279.5
**PHB-NC Ar/N2 (E30)**	29.8 ± 0.5	1309 ± 55	268.9	282.5
**PHB-NC Ar/NH3**	29.2 ± 0.9	1402 ± 55	260.8	273.0
**PHB-NC Ar-ACN**	30.6 ± 0.3	1408 ± 22	269.1	281.9
